# Assessment of Number of Critical Satellites for Ground-Based Augmentation System Continuity Allocation to Support Category II/III Precision Approaches

**DOI:** 10.3390/s23198273

**Published:** 2023-10-06

**Authors:** Junesol Song, Carl Milner

**Affiliations:** 1 Department of Mechanical Engineering, University of Suwon, Hwaseong-si 18323, Republic of Korea; 2 École Nationale de l’Aviation Civile (ENAC), 31400 Toulouse, France

**Keywords:** dual-frequency GBAS, number of critical satellites, Galileo

## Abstract

The ground-based augmentation system (GBAS) is a regional system supporting navigation and ensuring the integrity of aircraft near airports during precision approaches. Standardized at the international level, GBAS Approach Service Types (GASTs) C and D, which are defined for the GPS L1 signal, support CAT I and II/III precision approaches with decision heights of 200 and 50 ft, respectively. However, the future GBAS, GAST E, which utilizes dual-frequency and multi-constellation signals, and the GAST D1, defined for both GPS L1 and Galileo E1 signals, require the establishment of standards. To define the continuity requirement, the number of critical satellites must be considered. Currently, there is a lack of analysis on the number of critical satellites for various GBAS service types available to the public. This paper aims to evaluate the number of critical satellites for future GBAS service types, employing optimized GPS and Galileo constellations and assessing all potential protection levels worldwide. The methodology to model the difference of position solutions using the 30 s and 100 s smoothing filters is presented in detail to compute the protection level for GASTs D and D1. The resulting number of critical satellites can be used to define the continuity allocation of future GBAS.

## 1. Introduction

The ground-based augmentation system (GBAS) is a safety-critical system that provides correction and integrity information to airborne users in proximity to the airport. The importance of GBAS is its ability to support precision approaches. Among other augmentation systems based on Global Navigation Satellite System (GNSS), GBAS is currently the only one that supports precision approaches as low as 50 ft in decision height (DH) in terms of accuracy and integrity performance. If an aircraft determines that an approach or a landing cannot be executed successfully, it must initiate a missed approach or go around. In civil aviation, the DH refers to the minimum height at which the missed approach must be initiated during a precision approach [1]. For the aircraft to safely perform the missed approach, a navigation system supporting precision approach with a lower DH requires more stringent accuracy and integrity requirements. The precision approach is classified into Categories (CATs) I, II and III, enabling DHs of 200, 100 and 50 ft, respectively. Furthermore, GBAS supports CAT I, II and III precision approaches [2]. The GBAS Approach Service Type (GAST) C is a GBAS service type that supports CAT I precision approaches, utilizing an L1 single-frequency GPS signal. It is worth noting that the modification is applied in practical use, as can be seen, for example, at Frankfurt Airport, which operates CAT II with a DH of 100 ft using the GAST C station, despite the recommendations of the standards. Originally, GAST D was based on an L1 single-frequency GPS, and it supported CAT II/III precision approaches. The GASTs C and D were standardized by the ICAO [2,3]. The future GBAS service type that will utilize dual-frequency and multi-constellation signals and support CAT II/III is referred to as GAST E in accordance with the recent GBAS naming convention. Furthermore, GAST D has recently evolved into GAST D1 to indicate GBAS support for CAT II/III with both GPS L1 and Galileo E1 signals. It is used as a fallback mode from GAST E in case of loss of GPS L5 or Galileo E5 signal [4,5]. These future GBAS service types are currently under international discussion for standardization [4,5,6].

**Figure 1:**
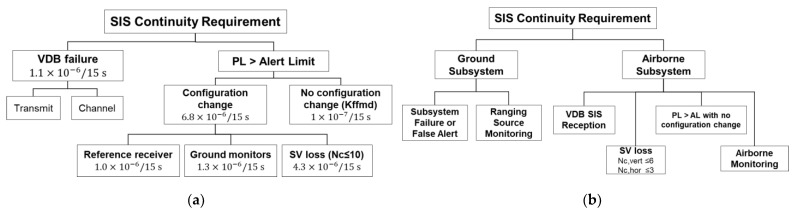
Continuity allocation: (**a**) GAST C [7]; (**b**) GAST D [3].

To define the continuity and integrity allocation for GASTs D1 and E, it is necessary to refer to the conventional GASTs C and D. Continuity is defined as the probability of losing Signal-In-Space (SIS) availability in the navigation system due to unscheduled interruptions [2]. Therefore, all possible components constituting the GBAS system that could cause unscheduled interruptions should be taken into account, and the total SIS continuity should be distributed among all components. Figure 1 illustrates the continuity allocation for GASTs C [7] and D [3]. Although the detailed classifications differ, both continuity allocations include factors such as ground subsystem failure (VDB failure), reference receivers, ground monitors, cases when the protection level (PL) exceeds the alert limit, and satellite or ranging source loss. Ranging source loss can occur due to satellite tracking loss, unscheduled satellite maneuvers or a satellite being set as unhealthy [3]. The probability (defined per time interval) of the ranging source loss can be calculated by multiplying the outage rate with the number of critical satellites. The outage rate represents the inverse of the Mean Time Between Outages (MTBO), which is conservatively assumed to be 9740 h [8]. A critical satellite is one whose loss (or exclusion due to monitor alert) results in a loss of continuity [7]. When critical satellites are included, the PL is below the alert limit (AL). Conversely, when critical satellites are excluded, the PL exceeds AL, requiring the operation to be aborted. Thus, the number of critical satellites plays a crucial role in conservatively finalizing the continuity allocations. Therefore, the number of critical satellites should be assessed to define the continuity allocation of the future GBAS service types such as GAST D1 with Galileo E1 and GAST E.

Currently, there is a lack of thorough analyses quantifying the number of critical satellites available to the public for various GBAS service types. Pullen [7] and the ICAO paper [3] present the numbers of critical satellites for GAST C and GAST D, respectively. However, in [3], only the upper bound for the number of critical satellites is provided, with no information on the assessment conditions. Kline et al. [9] assessed the availability of GBAS supporting CATs I, II and III based on the number of critical satellites. However, their study did not directly assess the number of critical satellites but rather analyzed the impact of the number of critical satellites on GBAS availability. Moreover, the divergence of the position errors from a 30 s and 100 s smoothing time constant is not considered in the protection level computation. Zhai et al. [10] conducted an analysis of the availability of Advanced Receiver Autonomous Integrity Monitoring (ARAIM) to support Required Navigation Performance (RNP) 0.1 and 0.3. Their analysis considered the continuity risk associated with the critical satellite dynamically, without pre-allocating a defined continuity risk for satellite loss. However, since ARAIM is not a ground-based augmentation system and its support in flight operation differs, the proposed approach may not be directly applicable to GBAS. Previous studies regarding the number of critical satellites are summarized in Table 1. Nevertheless, there are no publicly available evaluations of the number of critical satellites for future GBAS service types following the same continuity allocation criteria as defined for GAST C and D. Therefore, the purpose of this paper is to assess the number of critical satellites for the future GBAS service types GAST D1 and GAST E to facilitate the determination of the continuity allocation. The assessment will involve determining the PL of GASTs D1 and E. In particular, for GASTs D and D1, we will consider the divergence resulting from the use of 30 s and 100 s carrier-smoothing filters and provide a methodology for modeling it. Using the optimized GPS and Galileo constellations with 24 slots, we will individually exclude each visible satellite, then check the resultant PL against the alert limit to identify any potential critical satellites.

## 2. GBAS Protection Level

To assess the number of critical satellites, it is necessary to calculate the PL. The PL represents the confidence bound of the position error required to meet the integrity requirement. The computation of PL can be found in the ICAO [2] and RTCA standards [8]. This section briefly summarizes the PL computation and provides detailed descriptions of the components that constitute the PL, such as the divergence resulting from the use of the 30 s and 100 s carrier-smoothing filters.

### 2.1. GBAS Protection Level Equations

In GBAS, the computation of the PL involves two hypotheses regarding the fault condition: the fault-free hypothesis indicating the nominal condition, denoted as H0, and the single-fault hypothesis indicating a single ground receiver failure, denoted as H1. Typically, the PL is determined as the maximum value among the PLs of H0 and H1 as the following equations:

(1) $$LPL=\max\left\{LPL_{H0},LPL_{H1}\right\}$$

(2) $$VPL=\max\left\{VPL_{H0},VPL_{H1}\right\}$$

The symbols of the LPL and VPL denote the lateral protection level and vertical protection level, respectively. Firstly, the equation to calculate the PLs for the H0 hypothesis is expressed as the following, which are defined in the ICAO [2] and RTCA standards [8]:

(3) $$LPL_{H0}=K_{ffmd}\sigma_{lat}+D_{L}$$

(4) $$VPL_{H0}=K_{ffmd}\sigma_{vert}+D_{V}$$

where

$$\sigma_{lat}=\sqrt{\sum_{i=1}^{N}s_{lat,i}^{2}\sigma_{i}^{2}},\;\sigma_{vert}=\sqrt{\sum_{i=1}^{N}s_{vert,i}^{2}\sigma_{i}^{2}},\;\sigma_{i}^{2}=\sigma_{gnd,i}^{2}+\sigma_{tropo,i}^{2}+\sigma_{iono,i}^{2}+\sigma_{air,i}^{2}$$

The symbols $\sigma_{lat}$ and $\sigma_{vert}$ represent the standard deviations of the lateral and vertical position errors, respectively. These two terms can be computed from the sum of the vertical and lateral components of the projection matrix ($s_{lat,i}\;and\;s_{vert,i}$) multiplied with the standard deviation of the error in the differential range of each satellite ($\sigma_{i}$). The symbols $\sigma_{gnd},\;\sigma_{tropo},\;\sigma_{iono}$ and $\sigma_{air}$ represent the standard deviation of the smoothed ground receiver noise and multipath, the tropospheric residual, the ionospheric residual, and the airborne receiver noise and multipath, respectively. The projection matrix S can be computed by ${\left(H^{T}WH\right)}^{-1}H^{T}W$, where H and W are observation and weighting matrices, respectively. When considering a single constellation, S is a 4 by n matrix, where n is the number of visible satellites. The first three rows of S represent the x-, y- and z-axis components, denoted as $s_{x,i},\;s_{y,i}$ and $s_{z,i}$ for the i-th column (i-th satellite). The lateral and vertical projection components, $s_{lat,i}$ and $s_{vert,i}$ are defined as $s_{y,i}$ and $s_{z,i}+s_{x,i}\tan GPA$, respectively. Here, GPA indicates the glide path angle, which can be set to 2.5 degrees [3].

The LPL and VPL computed for the H1 hypothesis can be expressed as the following equations, which are defined in the standards [2,8]:

(5) $$LPL_{H1}=\max\left\{LPL_{j}\right\}+D_{L}$$

(6) $$VPL_{H1}=\max\left\{VPL_{j}\right\}+D_{V}$$

where

$$LPL_{j}=\left|B_{lat,j}\right|+K_{md}\sigma_{lat,H1}.$$

$$VPL_{j}=\left|B_{vert,j}\right|+K_{md}\sigma_{vert,H1}$$

$$\sigma_{lat,H1}=\sqrt{\sum_{i=1}^{N}s_{lat,i}^{2}\sigma_{i,H1}^{2}},\;\sigma_{vert,H1}=\sqrt{\sum_{i=1}^{N}s_{vert,i}^{2}\sigma_{i,H1}^{2}}$$

$$\sigma_{i,H1}^{2}={\left(\frac{M_{i}}{M_{i}-1}\right)}^{2}\sigma_{gnd,i}^{2}+\sigma_{tropo,i}^{2}+\sigma_{iono,i}^{2}+\sigma_{air,i}^{2}$$

Typically, the GBAS ground system consists of two to four reference receivers, and the pseudorange corrections generated at each reference receiver are averaged to further reduce the noise in the smoothed pseudorange. The standard deviation of the averaged noise is denoted as $\sigma_{gnd,i}$ under the fault-free condition. However, when considering the H1 hypothesis, which assumes that a single reference receiver is faulty and excludes one hypothetical faulty reference receiver, the standard deviation of the noise should be inflated by a factor of $\frac{M_{i}}{M_{i}-1}$. Here, $M_{i}$ denotes the number of reference receivers used to compute the correction for the i-th satellite [2,8]. Consequently, the standard deviation of the differential pseudorange residual increases, and it is denoted as $\sigma_{i,H1}$. This, in turn, results in an increased standard deviation of the lateral and vertical position errors, denoted as $\sigma_{lat,H1}$ and $\sigma_{vert,H1}$.

The terms $B_{lat,j}$ and $B_{vert,j}$ in Equations (5) and (6) represent the lateral and vertical B-values, respectively. These values can be obtained by transforming the range domain B-value into the position domain through multiplication by the projection matrix S. The range domain B-value is used to determine if the j-th reference receiver is faulty [2,8]. In GBAS, the ground system broadcasts the B-values of each satellite via a Type 1 Message [11]. The standard deviation of the B-value has been proposed by Shively et al. [12] for the PL simulation, as indicated by the following equation.

(7) $$\sigma_{B,vert}=\sqrt{\sum_{i=1}^{N}s_{vert,i}^{2}\frac{\sigma_{gnd,i}^{2}}{M_{i}-1}}\;$$

It is important to note that a 30 s time constant is used in the smoothing filter for GASTs D and D1, while GAST E could potentially employ a variable smoothing time constant up to 600 s [4]. Currently, for GAST E, an extension to the 600 s time constant is under discussion at the international level. In our paper, a 100 s time constant is used to test the dual-frequency dual-constellation GBAS to address conservative analysis. In particular, when GAST D is active, the two different position solutions are computed based on the 30 s and the 100 s smoothed pseudoranges [11]. The impact of using two different smoothing filters is reflected through the terms $D_{V}$ and $D_{L}$ in Equations (3)–(6). Since GAST D1 is not standardized currently, it is assumed that the same requirement as GAST D is applied to GAST D1. The methodology of computing these two terms will be detailed in the following subsection.

### 2.2. Consideration of D_V_ and D_L_ for GASTs D and D1

As mentioned earlier, when GASTs D and D1 are active, the two different position solutions are computed based on the 30 s and 100 s smoothed pseudoranges by receiving corrections from Type 11 and Type 1 Messages, respectively [11]. The terms $D_{V}$ and $D_{L}$ represent the difference between these two position solutions. However, in other iterations such as GASTs C and F, $D_{V}$ and $D_{L}$ are set to zero.

The non-zero values of $D_{V}$ and $D_{L}$ arise due to two factors that differentiate the outputs from the 30 s and 100 s smoothing [11]. The first factor is the impact of ionospheric delay, while the second factor is the impact of noise and multipath. The subsequent subsection will focus on computing the standard deviation associated with these impacts.

#### 2.2.1. The Impact of the Ionospheric Delay

The purpose of the smoothing filter in GBAS is to reduce noise error in the pseudorange residual. However, in the case of GASTs D and D1, which utilize single-frequency measurements, the input of the smoothing filter includes the ionospheric delay, resulting in an additional bias error, denoted as a filter add-up error [13]. Shively et al. [12] provide the equation to compute the D of the i-th satellite, denoted as $D_{R}^{i}$, as follows:

(8) $$D_{R}^{i}=-Obliquity(\theta^{i})\times G\times 2\times (\tau_{2}-\tau_{1})\times V_{air}$$

where, the function $Obliquity(\theta^{i})$ is used to compute the obliquity factor of the i-th satellite based on its elevation angle, $\theta^{i}$. The terms G, $\tau_{1}$, $\tau_{2}$ and $V_{air}$ represent the spatial gradient of the vertical ionospheric delay, the 30 s and the 100 s time constants for the carrier-smoothing filters, and the speed of the aircraft, respectively. Therefore, the standard deviation of $D_{R}^{i}$ can be expressed as shown in Equation (9).

(9) $$\sigma_{D_{R}^{i}}=Obliquity(\theta^{i})\times \sigma_{G}\times 2\times (\tau_{2}-\tau_{1})\times V_{air}.$$

In our analysis, $\sigma_{G}$ is set to 4 mm/km [14,15] and 8 mm/km [8]. The term $V_{air}$ is set to 161 kts (82.83 m/s) for the CAT III precision approach [16].

#### 2.2.2. The Impact of the Noise and Multipath

**Figure 2:**
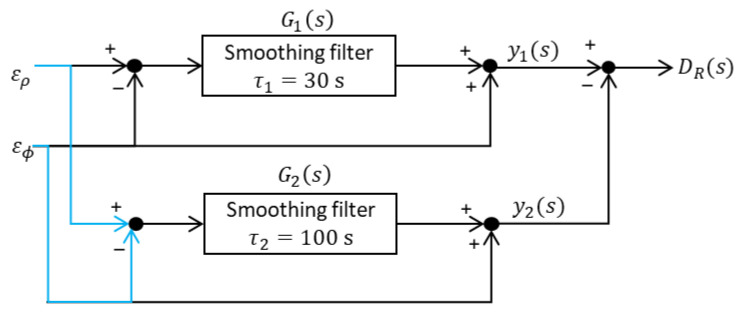
Block diagram to compute the noise and multipath in $D_{R}^{i}$.

To account for the impact of noise and multipath in $D_{V}$ and $D_{L}$, the standard deviations of these two errors need to be computed. To facilitate the computation of this term, we have constructed the following block diagram shown in Figure 2 which takes the noise and multipath of the pseudorange and carrier phase as inputs, denoted as $\varepsilon_{\rho }$ and $\varepsilon_{\phi }$, respectively. The noise and multipath in the smoothed pseudorange, obtained after applying two different smoothing filters, are denoted as $y_{1}$ and $y_{2}$, respectively. The difference between $y_{1}$ and $y_{2}$ is defined as $D_{R}$.

In Figure 2, the transfer functions of the two smoothing filters can be expressed as the following equation.

(10) $$G_{1}\left(s\right)=\frac{1}{\tau_{1}s+1},\;\;\;G_{2}\left(s\right)=\frac{1}{\tau_{2}s+1}.$$

**Figure 3:**
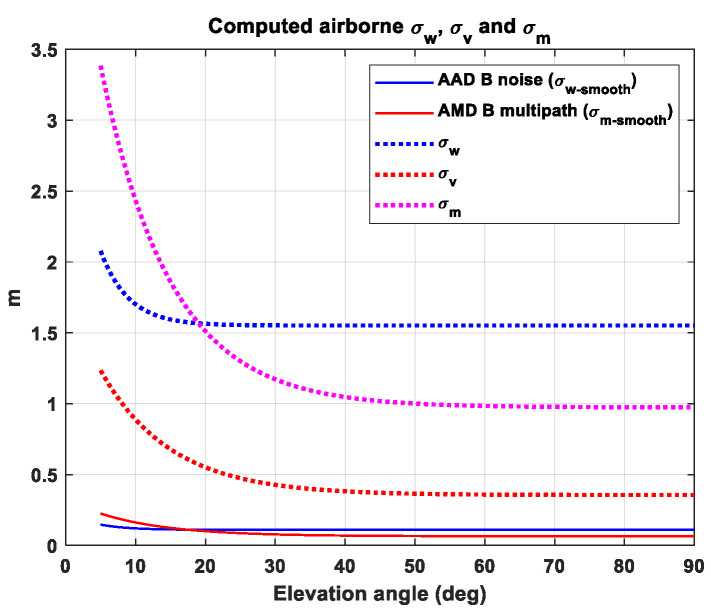
Comparison of standard deviations of smoothed noise ($\sigma_{w-smooth}$) and multipath ($\sigma_{m-smooth}$) defined in the standards and the estimated standard deviations of raw WGN ($\sigma_{w}$), multipath ($\sigma_{m}$) and process noise ($\sigma_{v}$) of the first-order GM model for the multipath.

Using Equation (10), the transfer function of the total system shown in Figure 3, when the input is defined as $\varepsilon_{\rho }\left(s\right)-\varepsilon_{\phi }\left(s\right)$, can be expressed as the following equation:

(11) $$G\left(s\right)\equiv G_{1}\left(s\right)-G_{2}\left(s\right)=\frac{(\tau_{2}-\tau_{1})s}{\left(\tau_{1}s+1)(\tau_{2}s+1\right)}.$$

To simplify the problem, we assume that the noise and multipath of the pseudorange measurement are significantly larger than those of the carrier phase measurement. In other words, $\varepsilon_{\rho }-\varepsilon_{\phi }\approx \varepsilon_{\rho }$. It is important to note that RTCA DO-245A [8] provides the noise and multipath model of the airborne receiver when the carrier-smoothing with a 100 s time constant is applied. The noise and multipath in the raw pseudorange measurement are denoted as w and m, respectively, and $\varepsilon_{\rho }=w+m$.

To compute the standard deviation of $D_{R}$, the standard deviation of the noise and multipath in the raw pseudorange measurement, denoted as $\sigma_{w}$ and $\sigma_{m}$, respectively, should be known. When the input is white Gaussian noise and the term $\sigma_{w}$ is known, the theoretical standard deviation of the smoothed noise can be easily obtained as a function of the smoothing time constant, as shown in Appendix A. When the input is the temporally correlated error such as multipath, Blanch et al. [17] derived a theoretical equation for the standard deviation of $y_{1}$ or $y_{2}$ as a function of the time constant of the smoothing filter and the time constant of the multipath. By utilizing these theoretical equations from Appendix A and [17], and models of the noise and the multipath in the smoothed pseudorange residual provided by RTCA DO-245A [8], we can estimate the standard deviation of the noise and multipath in the raw pseudorange measurement. This estimated information, combined with the transfer function G(s) in Equation (11), allows us to estimate the standard deviation of $D_{R}$.

To detail the procedure for computing the standard deviation of $D_{R}$, we derive the autocorrelation function of $D_{R}$ for two types of inputs: one is the case when the input is white Gaussian noise, and the other is the case when the input is a multipath, which is modeled as the first-order Gauss–Markov process.

- Input: White Gaussian Noise (WGN)

The autocorrelation of the input and its Laplace transformation, namely the power spectral density, can be expressed as the following equation:

(12) $$R_{w}\left(\tau \right)=\sigma_{w}^{2}\delta \left(\tau \right)\;$$

(13) $$S_{w}\left(s\right)=L\left\{R_{w}\left(\tau \right)\right\}=\sigma_{w}^{2}$$

The symbol $\sigma_{w}$ represents the standard deviation of the noise in the raw pseudorange measurement, w. The function δ indicates the Dirac delta function, and the term τ denotes the time lag. The symbol L represents an operator for Laplace transformation. The Laplace transformation of the autocorrelation of the output $D_{R}$ can be computed using the transfer function, G(s), and the power spectral density of the input, $S_{w}$, as follows [18]:

(14) $$L\left\{R_{D_{R}}\left(\tau \right)\right\}=S_{D_{R}}\left(s\right)=G\left(-s\right)S_{w}\left(s\right)G\left(s\right)=\sigma_{w}^{2}\left[\frac{\tau_{2}-\tau_{1}}{\tau_{2}+\tau_{1}}\cdot \frac{1}{1-\tau_{1}^{2}s^{2}}-\frac{\tau_{2}-\tau_{1}}{\tau_{2}+\tau_{1}}\cdot \frac{1}{1-\tau_{2}^{2}s^{2}}\right].$$

The power spectral density $S_{D_{R}}\left(s\right)$ can be transformed to the frequency domain by replacing s to jω, where ω represents the angular frequency. The variance of $D_{R}$ at steady state can be calculated by integrating the power spectral density of $D_{R}$, $S_{D_{R}}\left(j\omega \right)$, from ω=−∞ to ω=+∞ as follows [18]:

(15) $$Var\left\{D_{R}\right\}=\sigma_{R}^{2}=\int_{-\infty }^{\infty }S_{D_{R}}\left(j\omega \right)d\omega =\sigma_{w}^{2}\frac{{\left(\tau_{2}-\tau_{1}\right)}^{2}}{\tau_{1}\tau_{2}(\tau_{2}+\tau_{1})}.$$

It should be noted that the variance of $D_{R}$ is computed for each satellite.

- Input: first-order Gauss–Markov (GM) process

The time-correlated error, such as multipath, can be modeled as a first-order GM process using the following equation:

(16) $$m\left(k\right)=\beta m\left(k-1\right)+v(k).$$

The symbols k and v represent an epoch index and the process noise, whose standard deviation is denoted as $\sigma_{v}$. The relationship between $\sigma_{m}$ and $\sigma_{v}$ can be defined as $\sigma_{v}=\sigma_{m}\sqrt{1-\beta }$ [17]. The symbol β is defined as $e^{-\frac{∆t}{\tau_{mp}}}$, where $\tau_{mp}$ and ∆t represent the time constant of the multipath and the sampling interval, respectively. In the case of the multipath input, the autocorrelation of the input and its Laplace transformation can be expressed as the following equation:

(17) $$R_{m}\left(\tau \right)=\sigma_{m}^{2}e^{-\frac{\tau }{\tau_{mp}}}$$

(18) $$\;S_{m}\left(s\right)=L\left\{R_{m}\left(\tau \right)\right\}=\frac{2\sigma_{m}^{2}\tau_{mp}}{1-\tau_{mp}^{2}s^{2}}$$

The symbol $\sigma_{m}$ represents the standard deviation of the multipath at the steady state. The time constant of the multipath, $\tau_{mp}$, is set to 7 s [19]. The Laplace transformation of the autocorrelation of the output $D_{R}$ can be computed as follows [18]:

(19) $$L\left\{R_{D_{R}}\left(\tau \right)\right\}=S_{D_{R}}\left(s\right)=G\left(-s\right)S_{m}\left(s\right)G\left(s\right)\;=2\sigma_{m}^{2}{\tau_{mp}\left(\tau_{2}-\tau_{1}\right)}^{2}\cdot \left[\frac{\tau_{1}^{2}}{\tau_{1}^{2}-\tau_{mp}^{2}}\cdot \frac{1}{1-\tau_{1}^{2}s^{2}}-\frac{\tau_{2}^{2}}{\tau_{2}^{2}-\tau_{mp}^{2}}\cdot \frac{1}{1-\tau_{2}^{2}s^{2}}+\frac{\tau_{mp}^{2}{\left(\tau_{2}-\tau_{1}\right)}^{2}}{\left(\tau_{1}^{2}-\tau_{mp}^{2})(\tau_{2}^{2}-\tau_{mp}^{2}\right)}\cdot \frac{1}{1-\tau_{mp}^{2}s^{2}}\right]$$

By integrating the power spectral density of $D_{R}$, the variance of $D_{R}$ at steady state can be calculated using the same approach shown in Equation (15):

(20) $$Var\left\{D_{R}\right\}=\sigma_{R}^{2}=\int_{-\infty }^{\infty }S_{D_{R}}\left(j\omega \right)d\omega =\sigma_{D_{R}}^{2}=2\sigma_{m}^{2}\tau_{mp}\cdot \frac{\left(\tau_{2}-\tau_{1}\right)\left\{\tau_{mp}\left(\tau_{mp}+\tau_{2}-\tau_{1}\right)+\tau_{1}\tau_{2}\right\}}{\left(\tau_{1}^{2}-\tau_{mp}^{2})(\tau_{2}^{2}-\tau_{mp}^{2}\right)}.$$

It should be noted that the variance of $D_{R}$ is computed for each satellite.

- Determination of $\sigma_{w}^{2}$ and $\sigma_{m}^{2}$ to compute the variance of $D_{R}$

For both cases of white Gaussian noise and the first-order Gauss–Markov process as input, the terms $\sigma_{w}^{2}$ and $\sigma_{m}^{2}$ in Equations (15) and (20) should be known to compute the standard deviation of $D_{R}$.

As mentioned earlier, carrier smoothing is employed to mitigate the stochastic errors present in the pseudorange measurements. In the case of white Gaussian Noise (WGN), which exhibits no time correlation, the standard deviation of the smoothed noise can be expressed as the following equation:

(21) $$\sigma_{w-smooth}^{2}=\sigma_{w}^{2}\frac{1}{K^{2}}\frac{1}{1-A^{2}}.$$

The detailed derivation can be found in Appendix A. The RTCA DO-245A [8] also defines the standard deviation of the smoothed noise error, and the Airborne Accuracy Designator (AAD) B is chosen for use of $\sigma_{w-smooth}^{2}$ [8]. This information allows for the determination of the term $\sigma_{w}^{2}$ in Equation (21), which is then substituted into Equation (15) to compute the standard deviation of $D_{R}$.

Blanche et al. [17] derived the theoretical equation for the smoothed multipath error, as modeled as in Equation (16). The standard deviation of the smoothed multipath error can be expressed as the following [17]:

(22) $$\sigma_{m-smooth}^{2}=A^{2k}\left\{\frac{1+\beta }{K(1-\beta )}-\frac{2\beta }{K^{2}{\left(1-\beta \right)}^{2}}(1-\beta^{K})\right\}+\frac{\beta A^{2k-1}}{K^{2}}\left\{\frac{1-{\left(A\beta \right)}^{k}}{1-A\beta }\frac{1-\beta^{K}}{1-\beta }\right\}+\;\frac{1}{K^{2}}\left[\frac{1-A^{2k}}{1-A^{2}}+\frac{2}{1-{\left(\frac{A}{\beta }\right)}^{2}}\left\{\frac{1-{\left(\beta A\right)}^{k}}{1-\beta A}-\frac{1-A^{k}}{1-A^{2}}\right\}\right]\sigma_{v}^{2}.$$

In Equation (22), the symbol *k* represents the epoch index. The steady-state value of Equation (22) can be obtained by letting *k* approach infinity, resulting in the following equation:

(23) $$\sigma_{m-smooth}^{2}=\frac{1}{K^{2}}\left[\frac{1}{1-A^{2}}+\frac{2}{1-{\left(\frac{A}{\beta }\right)}^{2}}\left\{\frac{1}{1-\beta A}-\frac{1}{1-A^{2}}\right\}\right]\sigma_{v}^{2}.$$

In Equation (23), the symbols K and A denote $\frac{\tau_{sm}}{∆t}$ and $1-\frac{1}{K}$, respectively, where $\tau_{sm}$ represents the time constant of the carrier-smoothing filter (i.e., 30 s for GAST D and D1, and 100 s for GAST E). The RTCA DO-245A [8] provides the standard deviation of the smoothed range error after applying the smoothing filter with a 100 s time constant at the airborne receiver, which corresponds to the term $\sigma_{m-smooth}$ in Equation (23). In our analysis, the Airborne Multipath Designator (AMD) B is chosen for the airborne model, as shown in Table 2. By substituting the value of AAD B receiver model to $\sigma_{m-smooth}$ in Equation (23), $\sigma_{v}$ and $\sigma_{m}$ can be determined accordingly using the relationship $\sigma_{v}=\sigma_{m}\sqrt{1-\beta }$. This term is substituted to Equation (20) to compute the standard deviation of $D_{R}$. Figure 3 represents the computed $\sigma_{w}$, $\sigma_{v}$ and $\sigma_{m}$ using the approaches described above.

**Figure 4:**
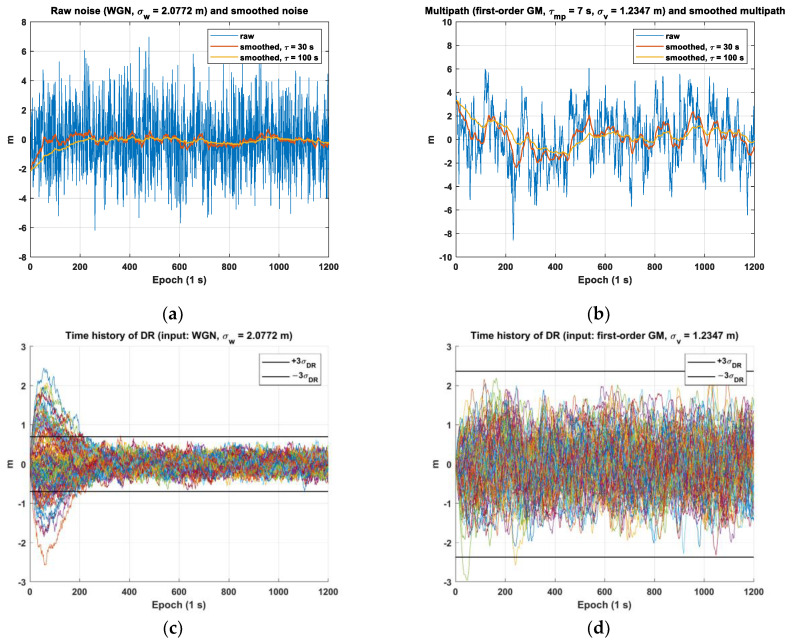
Validation of the derived standard deviations of $D_{R}$ for noise and multipath inputs: (**a**) raw and smoothed noises; (**b**) raw and smoothed multipaths; (**c**) time history of $D_{R}$ for noise with a 3-sigma bound of $\sigma_{DR}$; (**d**) time history of $D_{R}$ for multipath inputs with a 3-sigma bound of $\sigma_{DR}$.

To validate the theoretically derived standard deviation of $D_{R}$ as shown in Equations (15) and (20), we conducted simulations by generating raw noise and multipath data. We implemented the time-invariant first-order smoothing filter and applied this filter to smooth the generated noise and multipath. In generating the raw noise and multipath, we relied on estimated standard deviations: $\sigma_{w}$ for raw noise, $\sigma_{m}$ for multipath, and $\sigma_{v}$ for process noise in the first-order GM process, as shown in Figure 4. These estimates were based on the smoothed noise ($\sigma_{w-smooth}$) and multipath ($\sigma_{m-smooth}$) values provided in the standards [8] at an elevation angle of 5 degrees. For multipath error, the time constant of the first-order GM process is set to 7 s [19]. The raw and smoothed noise and multipath are depicted in Figure 4a,b, respectively. In addition, we applied two different smoothing time constants of 30 s and 100 s to compute $D_{R}$ for both noise and multipath by subtracting the two smoothed values. Using Equations (15) and (20), the theoretical steady-state standard deviation of $D_{R}$ ($\sigma_{DR}$) is computed based on the values of $\sigma_{w}$, $\sigma_{m}$, and $\sigma_{v}$. Figure 4c,d show that the $D_{R}$, represented by colored lines for various simulation cases, can be bounded by the theoretically derived $\sigma_{DR}$ with a 99.7% (3-sigma) confidence level.

## 3. Methodology

**Figure 5:**
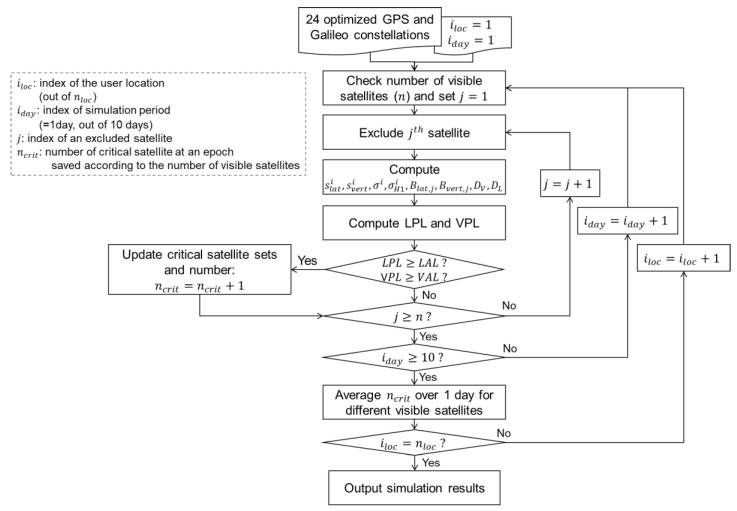
Block diagram to compute the noise and multipath in $D_{R}^{i}$.

The overall procedure for computing the number of critical satellites is illustrated in Figure 5. For the simulation, we utilize the optimized GPS [20] and Galileo constellations [21], which consist of 24 satellites for each constellation. These constellations are widely employed for the continuity and integrity monitoring simulations such as in GBAS, RAIM and ARAIM [22,23,24]. To account for satellite visibility variations, we set the location of the users over latitudes ranging from −85 to 85 degrees and longitudes ranging from −180 to 180 degrees, with a 5-degree interval. At each user position, we checked the visible satellites and their count. Subsequently, we sequentially exclude one visible satellite at a time and compute the LPL and VPL using Equations (1)–(6). We then compare the computed LPL and VPL with the lateral alert limit (LAL) and vertical alert limit (VAL), which are set to 17 m and 10 m [8], respectively. If the computed values exceed these limits, the excluded satellite is identified as a critical satellite, and the number of critical satellites ($n_{crit}$) is incremented by 1. We save the number of critical satellites separately based on the number of visible satellites. For each user location ($i_{loc}$), we repeat this procedure for a duration of 10 days to have a significant number of samples for averaging the number of critical satellites. After conducting the simulation for all user locations and over 10 days, the average number of critical satellites is computed for each number of visible satellites to facilitate the comparison with the results obtained in the previous study [7]. This is carried out to facilitate the comparison of the obtained results with those presented in [7], which contains the only publicly available data on the number of critical satellites. Reference [3] also provides a bound of the number of critical satellites for GAST D, although it lacks further details. The epoch interval is set to 30 min.

To compute the LPL and VPL using Equations (1)–(6), it is necessary to have the standard deviation models for the error sources. In our simulation, we adopted the models defined in RTCA DO-245A [8] for the ground receiver, airborne receiver, tropospheric and ionospheric delay residuals. We assume a total of four ground receivers in the simulation. Table 2 summarizes parameters of the error models used in the simulation.

In our simulation, we computed the number of critical satellites in two flight phases: one from the location where the decision height equals 200 ft to the threshold and the other from the threshold to roll out, as specified in [3]. The exposure times for these phases are 15 s each [3], as depicted in Figure 1. It should be noted that the number of critical satellites is computed at the initial stage of each operation period and is assumed to remain unchanged for the entire 15 s duration.

The numbers of critical satellites are computed for GAST D1 for both GPS L1 (previously denoted as GAST D) and Galileo E1 signals and GAST E based on the dual-frequency GPS/Galileo, respectively. In addition to the generic GAST E, single-frequency L1 and single constellation processing modes are considered, which could serve as a potential fallback mode from GAST E in the event of the loss of an L5 frequency or of one constellation. Furthermore, GAST E does not mandate the use of dual constellations.

## 4. Results

This section provides the simulation results of the number of critical satellites for GASTs D1 and E.

**Figure 6:**
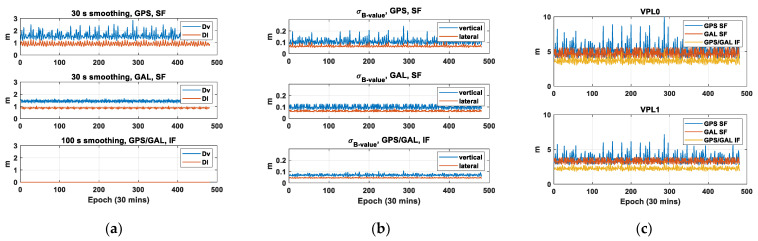
Results of the standard deviation of D_L_, D_V_, lateral and vertical B-values and corresponding VPL for GAST D1 with GPS L1, and D1 with Galileo E1 and F at the user location (latitude, longitude) = (45°, 0°): (**a**) D_L_ and D_V_; (**b**) lateral and vertical B-value; (**c**) VPL0 and VPL1.

**Figure 7:**
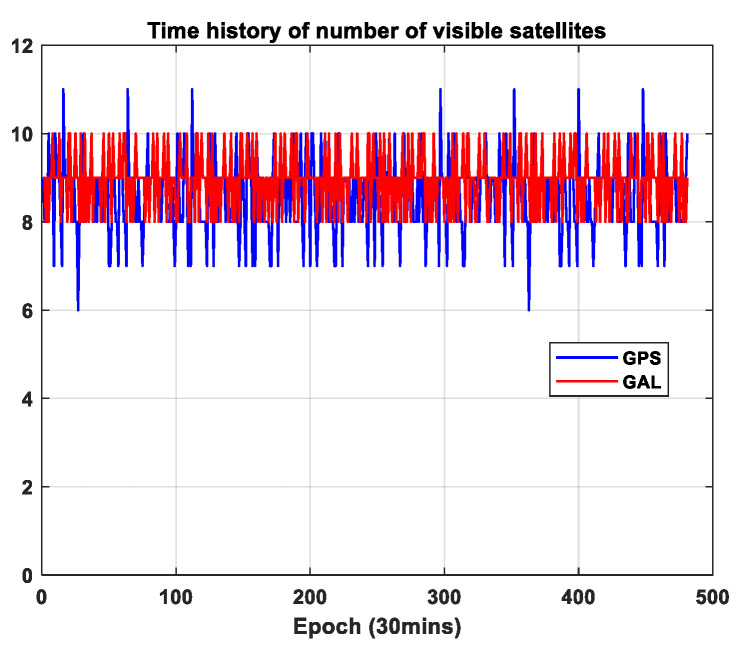
Time history of the number of visible satellites for GPS and Galileo at the user location (latitude, longitude) = (45°, 0°).

In case of GAST D1, the difference in the lateral and vertical position errors from 30 s and 100 s carrier-smoothing filters, denoted as $D_{L}$ and $D_{V}$, should be accounted for. The figures in the two upper panels of Figure 6a,b show the computed $D_{L}$, $D_{V}$, and lateral and vertical B-values. Figure 6c depicts the corresponding VPL. The values are computed for the user location (latitude, longitude) = (45°, 0°). The average value of each term for GAST D1 with GPS L1 and with Galileo E1 is at a similar level. In particular, the variation in the values is larger for GAST D1 with GPS L1 compared with GAST D1 with Galileo E1. It can be understood by comparing the time history of the number of visible satellites for GPS and Galileo, as shown in Figure 7. In Figure 7, the number of visible satellites decreases to 7 for GPS, while for Galileo, it remains above 8 throughout the simulation period. This is believed to be from the different orbit design of GPS and Galileo. To be more specific, Galileo satellites are in the orbital plane with a higher altitude and an inclination angle, which leads to an improvement in the satellite geometry compared with GPS [25]. In case of VPL, GAST E provides the smallest value: For VPL0, the mean values for GAST D1 with GPS L1, and D1 with Galileo E1 and E are 5.17 m, 4.73 m and 3.69 m, respectively, and for VPL1, the mean values are 3.63 m, 3.32 m and 2.26 m, respectively. Although the pseudorange noise can be inflated, associated with the linear combination of the measurements to form the ionosphere-free (IF) term, the use of GAST E is beneficial due to the larger performance gain brought by the use of multiple constellations.

**Figure 8:**
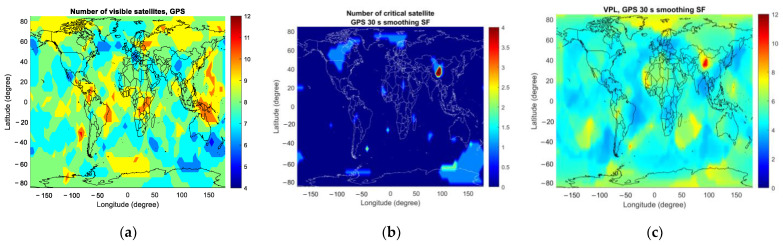
The number of visible satellites, the number of critical satellites and VPL for GAST D1 with GPS L1 at the initial epoch of the simulation: (**a**) number of visible satellites; (**b**) number of critical satellites; (**c**) VPL.

**Figure 9:**
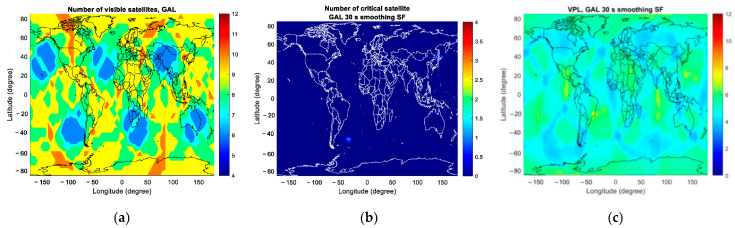
The number of visible satellites, the number of critical satellites and VPL for GAST D1 with Galileo E1 at the initial epoch of the simulation: (**a**) number of visible satellites; (**b**) number of critical satellites; (**c**) VPL.

At the first epoch of the simulation, Figure 8 and Figure 9 present the number of visible satellites, the number of critical satellites and VPL for GAST D1 with GPS L1 and D1 with Galileo E1 worldwide, respectively.

**Figure 10:**
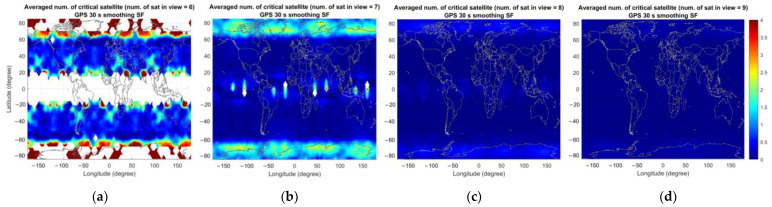

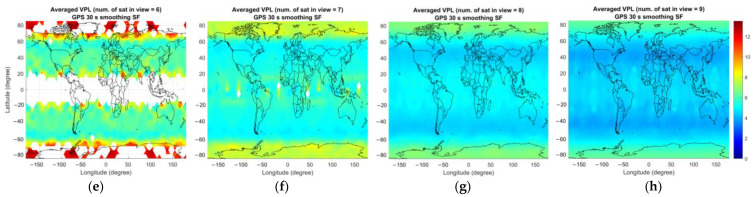
Averaged number of critical satellites (**upper** panel) and VPL (**lower** panel) for GAST D1 with GPS L1 over the simulation period for different numbers of visible satellites ($n_{vis}$): (**a**,**e**) $n_{vis}=6$; (**b**,**f**) $n_{vis}=7$; (**c**,**g**) $n_{vis}=8$; (**d**,**h**) $n_{vis}=9$.

**Figure 11:**
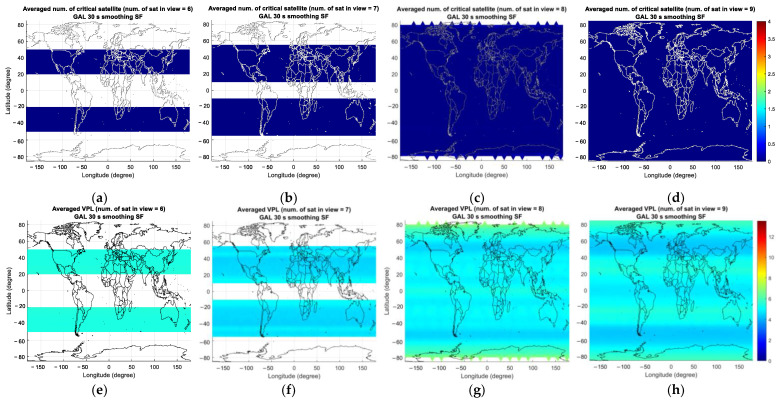
Averaged number of critical satellites (**upper** panel) and VPL (**lower** panel) for GAST D1 with Galileo E1 over the simulation period for different numbers of visible satellites ($n_{vis}$): (**a**,**e**) $n_{vis}=6$; (**b**,**f**) $n_{vis}=7$; (**c**,**g**) $n_{vis}=8$; (**d**,**h**) $n_{vis}=9$.

**Figure 12:**
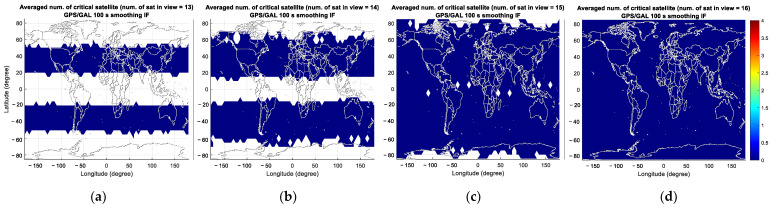

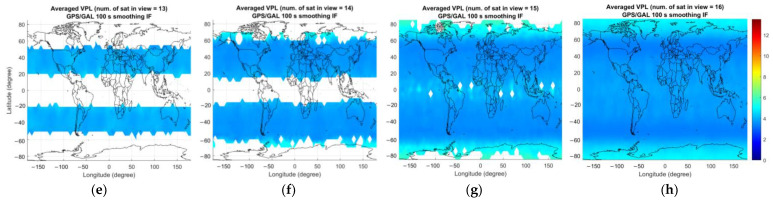
Averaged number of critical satellites (**upper** panel) and VPL (**lower** panel) for GAST E over the simulation period for different numbers of visible satellites ($n_{vis}$): (**a**,**e**) $n_{vis}=13$; (**b**,**f**) $n_{vis}=14$; (**c**,**g**) $n_{vis}=15$; (**d**,**h**) $n_{vis}=16$.

As mentioned previously, the computed number of critical satellites is saved separately according to the number of visible satellites and is averaged over the simulation period. Figure 10, Figure 11 and Figure 12 represent the average number of critical satellites and VPL for GASTs D1 and E. For GAST D1 with GPS L1 and D1 with Galileo E1, the results are shown for the number of visible satellites ranging from 6 to 9 and from 13 to 16 for GAST E. To facilitate the comparison among GBAS service types, the colormap axis is set to the same range for GASTs D1 and E. Note that the presented results are derived for $\sigma_{G}=4\;mm/km$ and the flight phase from the location where the decision height equals 200 ft to the threshold.

**Figure 13:**
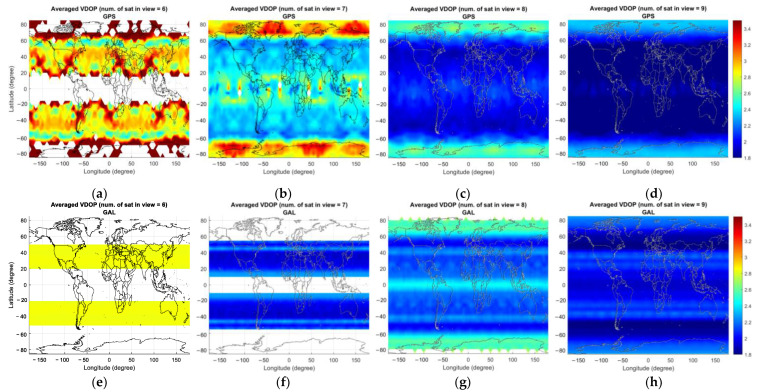
Averaged VDOP for GPS (**upper** panel) and Galileo (**lower** panel) for different numbers of visible satellites ($n_{vis}$): (**a**,**e**) $n_{vis}=6$; (**b**,**f**) $n_{vis}=7$; (**c**,**g**) $n_{vis}=8$; (**d**,**h**) $n_{vis}=9$.

In the case of GAST D1 with GPS L1, Figure 10a shows the number of critical satellites when there are six satellites in view. The colored region represents the area where any six GPS satellites are visible. The number of critical satellites is larger near the poles and the equator due to poor satellite geometry under the condition of the number of visible satellites being 6. This can be seen in Figure 13a–d, which represent the average vertical dilution of precision (VDOP) for GPS for different numbers of visible satellites. Accordingly, a relatively large VPL can be observed near the poles and the equator compared with at the mid-latitude region.

**Figure 14:**
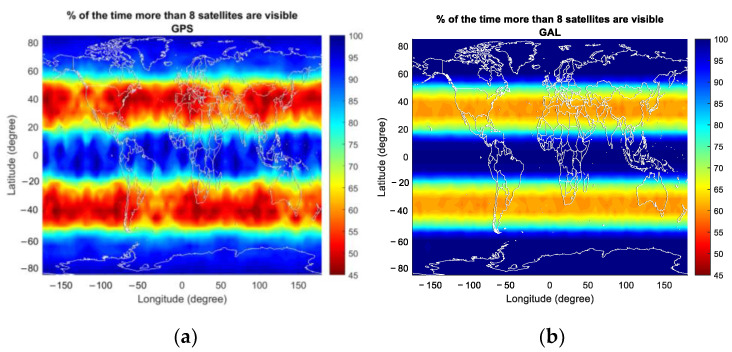
The percentage of the time when more than eight satellites are visible for (**a**) GPS; (**b**) Galileo.

In the case of GAST D1 with Galileo E1, the number of critical satellites is zero, unlike the GPS case where a non-zero number of critical satellites could be observed for six to nine visible satellites. This is due to the slightly improved satellite geometry for Galileo especially in the high latitude region, which can be seen in Figure 14, representing the average VDOP of GPS and Galileo for different numbers of visible satellites. In the high-latitude region, including the region near the pole, it is possible to observe more than eight satellites for Galileo, as shown in Figure 11g, resulting in an improved VPL for Galileo compared with that for GPS. We attribute this to the variation in the orbit design between GPS and Galileo. To be more specific, Galileo satellites are located in orbital planes with a slightly higher altitude and inclination angle compared with that of GPS satellites, which improves the satellite geometry in the high-latitude region [25]. Figure 14 presents the percentage of the time when more than eight satellites are visible for GPS and Galileo. The results are in line with the intent of Galileo orbit design, which improves the satellite geometry in the high-latitude region by extending the time span when a large number of satellites is visible. Satellite geometry is generally better for the mid-latitude region compared with the high-latitude region, which is reflected in Figure 13. The number of critical satellites for eight visible satellites is slightly larger than that for seven visible satellites. Up to seven visible satellites, the number of critical satellites cannot be computed near the pole region. When the number of visible satellites becomes eight, the number of critical satellites can be computed near the pole region and since the satellite geometry is poor near the pole compared with other regions, the computed number of critical satellites is slightly larger when the number of visible satellites is eight than when it is seven.

Figure 12 represents the average number of critical satellites and VPL for GAST E. Compared with the results of GAST D1 with GPS L1 and D1 with Galileo E1, the number of critical satellites is significantly reduced in GAST E due to the use of dual constellations (DC) of GPS and Galileo: the number of critical satellites is zero for 13 to 16 visible satellites.

Table 3 summarizes the number of critical satellites of GAST D1 with GPS L1 and D1 with Galileo E1 computed for each case of number of satellites in view, the phase of flight and the standard deviation of the vertical ionospheric gradient. In the case of GAST D1 with GPS L1, which indicates the GPS only single-frequency (SF) L1 processing mode, the number of critical satellites monotonically decreases as the number of visible satellites increases. This characteristic aligns with the findings from a previous study on the number of critical satellites for GAST C [6], which supports the CAT I precision approach. Unlike GAST D1 with GPS L1, for GAST D1 with Galileo E1, the number of critical satellites is almost zero for most cases in Table 3. As previously indicated in Figure 10, the computed VPL with eight satellites in view around the pole region is larger than the computed VPL around the mid-latitude region with only six or seven visible satellites due to the worse satellite geometry, as reflected in Figure 13. This explains why the number of critical satellites is non-zero for specific cases with seven to eight satellites, as indicated in Table 3.

As mentioned earlier, the remaining ionospheric delay in the SF pseudorange causes the filter add-up error in the smoothed measurement. Therefore, the number of critical satellites and the VPL are larger for a higher value of the standard deviation of the vertical ionospheric gradient, as shown in Table 3.

The number of critical satellites assessed for the lateral direction is not presented here because the assessed number is zero for all cases except when there are four satellites in view. This is due to the fact that the computed LPL does not exceed LAL of 17 m.

Table 4 and Table 5 present the number of critical satellites computed for GAST E when the standard deviation of the vertical ionospheric delay is set to 4 mm/km and 8 mm/km, as reported in references [8,14,15]. Since GAST E utilizes DC and dual frequencies (DF), there is a chance of a wide fault occurring on one constellation or the signals on a certain frequency not being available. In such a situation, GAST E should fall back to other GBAS service types, adjusting the GBAS processing to utilize available constellations and frequencies. To account for these scenarios, the simulation is conducted not only for a generic GAST E but also for GAST E using GPS/Galileo with SF mode and for GAST E using single-constellation (SC) GPS only with SF and DF modes. In the SF processing mode, we assumed that the airborne conducts ionosphere monitoring. This is because the ionospheric delay cannot be completely eliminated in the pseudorange, unlike in the case of the IF combination. Therefore, it is necessary to monitor the state of the ionospheric delay.

When only an SC is available, the number of critical satellites is assessed for both the DF and SF modes. The number of critical satellites is smaller for SF. Since the distance between the aircraft and the ground receivers is usually less than 10 km, the residual ionospheric delay becomes small, and its impact is not prominent even if the measurement combination is not employed. However, in DF mode, the noise in the IF measurements is a factor of 2.59 larger than that in the SF measurement [26]; thus, the performance is limited.

In the case of a generic GAST E, the number of critical satellites is almost zero for all scenarios of the number of satellites in view, except for five, which is the minimum number of satellites required for navigation. The number of critical satellites assessed using LPL is not presented because it results in zero for all cases, except when the number of visible satellites is the minimum required value for navigation.

By comparing the results of GAST D1 with GPS L1 and GAST E using GPS SF, we can understand the impact of the time constants of the carrier-smoothing filter. As mentioned previously, a 30 s time constant is used for GAST D1 and 100 s is used for GAST E. The larger the time constant of the smoothing filter, the more effective it is in reducing the noise error in the pseudorange in general. However, in the SF processing mode, the ionospheric delay is not completely eliminated in the measurement, and accordingly, it causes the filter add-up error, which is proportional to the time constant of the smoothing filter [13]. From the simulation results, a smaller number of critical satellites is observed for GAST E using GPS SF with the 100 s of the smoothing time constant compared with the GAST D1 with GPS L1, which uses the 30 s of the smoothing time constant. However, the number of critical satellites becomes similar when a larger standard deviation of the vertical ionospheric gradient is used because of the increased filter add-up error for GAST E using GPS SF. It should be noted that the standard deviation of the vertical ionospheric gradient has no impact on the dual-frequency measurements.

## 5. Discussion

In this paper, we have assessed the number of critical satellites, which are those whose loss leads to loss of continuity, for various GBAS service types, such as GAST D1 and E. The number of critical satellites is required to define the continuity allocation in GAST D1 with Galileo E1 and E, which have not yet been standardized like GASTs C and D (or D1 with GPS L1). GAST C assumes 10 critical satellites [7], while GAST D1 with GPS L1 utilizes 6 and 3 as the number of critical satellites for the vertical and lateral directions, respectively [3]. Currently, detailed information regarding the analysis of the number of critical satellites for GAST D1 with GPS L1 is not publicly available, and it has not been evaluated for GAST D1 with Galileo E1 and GAST E. To assess the number of critical satellites, the protection level should be computed. In GAST D1 with GPS L1 and D1 with Galileo E1, we should consider Dv and Dl, which correspond to the difference between position estimates based on a 30 s and 100 s smoothing filter. We propose the theoretical derivation to compute the standard deviation of Dv and Dl due to the noise and multipath from the standard deviation of the noise and multipath at the airborne receiver given in the standards. Using the proposed method, we compute the protection levels for the visible satellites by excluding one satellite at a time. Then, we compare each protection level with the alert limit. If the protection level with a particular excluded satellite exceeds the alert limit, the excluded satellite is determined to be a critical satellite [7,8,9,10]. The computation of the protection level is based on the error uncertainty models defined in the RTCA DO-245. Specifically, for GAST D1 with GPS L1 and D1 with Galileo E1, we should take into account the difference between the position solutions based on the 30 s and 100 s smoothing filters. The difference is associated with two error terms: the ionospheric delay and the noise and multipath. The ionospheric delay is modeled based on the reference paper, and the impact of the noise and multipath in the difference is modeled theoretically using the transfer functions of the two smoothing filters.

Comparing GAST D1 with GPS L1 and D1 with Galileo E1, the number of critical satellites is much larger for GAST D1 with GPS L1 than for GAST D1 with Galileo E1. In the case of GPS, the average number of critical satellites is mainly determined by the number near the pole region due to poor satellite geometry when the number of visible satellites is low. As the number of visible satellites increases, the DOP near the pole region decreases but remains higher than that in other regions. In contrast, for Galileo, thanks to the orbit design, more than seven satellites are visible near the pole region due to the orbit design. As a result, the number of critical satellites near the pole region cannot be computed when the number of visible satellites is less than eight. Therefore, the number of critical satellites is zero, except when there are eight or nine, allowing for the computation of critical satellites near the pole region. This observation is consistent with the improvement in satellite geometry achieved by the Galileo orbit design. Specifically, Galileo satellites are positioned in an orbital plane with a slightly higher altitude and inclination angle compared with GPS satellites, resulting in improved satellite geometry in the high latitude region [25].

Comparing the GAST D1 with GPS L1 with the GAST E using GPS-only SF, it can be seen that the use of a longer smoothing time constant results in a lower protection level and a reduced number of critical satellites. Even though the ionospheric delay is not completely eliminated in the SF differential pseudorange residual, the ionospheric residual is small due to the short baseline between the aircraft and the GBAS ground receiver. Further comparing GAST E using GPS-only DF with GAST E using GPS-only SF, the benefits of a dual-frequency signal are not prominent due to the inflation of noise and multipath errors in the IF measurement combination and the small ionospheric residual caused by the short baseline. It should be noted, however, that the performance of the GAST E using GPS-only SF may degrade under severe vertical ionospheric delay gradients. The use of dual constellations significantly improves the performance of the GAST E with DF processing mode when compared with the GAST E with GPS-only DF.

The current GAST D1 with GPS L1 (or GAST D) recommends using six and three of the number of critical satellites for the vertical and lateral directions, respectively [3]. The simulation results for GAST D1 with GPS L1 and D1 with Galileo E1 are consistent with this recommendation for the vertical direction, as shown in Table 3. In addition, the simulation results for GAST E are also consistent with the recommendation, providing sufficient margin, especially when dual constellations are used. As a result, a smaller number of critical satellites could be used to define the continuity allocation for satellite loss in GAST E compared with the number of critical satellites considered in GAST D1 with GPS L1. We expect that the assessment of the number of critical satellites presented in this paper could be used as a technical reference to determine the continuity allocations for the ranging source loss of the future GBAS service types.

## 6. Conclusions

This paper proposes the theoretical derivations of Dv and Dl, which represent the difference between position estimates in the lateral and vertical directions based on a 30 s and 100 s smoothing filter, respectively. This approach offers a method to determine the standard deviations of the raw noise and multipath, using the smoothed noise and multipath models defined in the standards. Using these derived values, we can compute the standard deviation of Dv and Dl using the proposed equation, eliminating the need for statistical analysis based on actual smoothed measurements. It is important to note that our theoretical derivation of Dv and Dl due to multipath assumes that the multipath can be modeled as a first-order GM process. However, the proposed approach may introduce errors if the time constant of the multipath is inaccurately estimated or if the multipath behaves significantly differently from the first-order GM process. Therefore, while our simulation provides valuable insight, it can be further validated by real measurements in the future to provide a more practical perspective on the obtained results.

In our simulation, we utilized the optimized 24-slot GPS and Galileo constellations. It is worth noting that the current operational GPS constellation consists of 27 slots, as confirmed by Official U.S. government information about the GPS [27]. This expanded constellation provides enhanced coverage and navigation performance compared with the one used in the simulation. Therefore, we believe that the simulation results presented in our paper represent worse-case scenarios compared with the currently deployed GPS and Galileo constellations. That is, the resulting number of critical satellites in this paper could serve as a bound for the number achievable in reality.

In our analysis, the number of critical satellites is evaluated under all-in-view conditions for GASTs D1 and E. The value obtained can be used to determine the continuity requirement for future GBAS solutions. In practice, there might be a high chance of losing satellite signals due to aircraft maneuvering and cycle slips. Therefore, an analysis of the impact of satellite loss on the number of critical satellites could be undertaken in the future.

## Figures and Tables

**Table 1:** List of references on the number of critical satellites (Nc: number of critical satellites).

References	Types of Augmentation System and Supported Approach	Remarks
Pullen [7]	GBAS, CAT I (CAST C)	- Nc assessed
ICAO SARPs [3]	GBAS, CAT II/III (GAST D)	- Nc assessed - Simulation condition not provided
Kline et al. [9]	GBAS; CATs I, II, III	- Nc not assessed - Only the impact of hypothetical Nc on the availability analyzed
Zhai et al. [10]	ARAIM, RNP 0.1 and 0.3	- Nc and corresponding continuity requirement of SV loss dynamically assessed and allocated - Not applicable to GBAS

**Table 2:** Parameter settings for the error models and the simulation scenario.

Flight Phase Layout		
Parameters	DH = 200 ft to Threshold	Threshold to Roll Out
Glide Path Angle (GPA)	2.5 degrees [3]	
$D_{th}$ (Distance from GND ^1^ to threshold)	5 km [16]	
Residual ionospheric uncertainty		
Parameters	DH = 200 ft to threshold	Threshold to roll out
$\sigma_{G}$ (Vertical ionosphere gradient)	4 mm/km [14,15] 8 mm/km [8]	
$x_{air}$ (Slant range distance from an aircraft location and a reference point)	$\frac{\left(200ft)\times (0.3048m/ft\right)}{\tan GPA}+D_{th}$	$D_{th}$
$v_{air}$ (Speed of the aircraft)	161 knots (82.83 m/s) [16]	
Residual tropospheric uncertainty		
Parameters	DH = 200 ft to threshold	Threshold to roll out
$\sigma_{N}$ (Refractivity uncertainty)	33 [16]	
$h_{0}$ (Tropospheric scale height)	15730 m [16]	
∆h (Difference of altitude between an aircraft and the GND ^1^ subsystem)	200 ft	0
Receiver error model		
Parameters	DH = 200 ft to threshold	Threshold to roll out
$\sigma_{air}$ (Airborne receiver model: AAD B, AMD B)	$RMS_{pr,air,GPS}\left(\theta \right)=0.11+0.13e^{-\frac{\theta }{4}}$ [8] $\sigma_{multipath}\left(\theta \right)=(0.13+0.53e^{-\frac{\theta }{10}})/2$ [8]	
$\sigma_{gnd}$ (Ground receiver model: GAD ^2^ C)	$RMS_{pr,gnd,GPS}\left(\theta \right)=\left\{\begin{array}{l} \sqrt{\frac{{\left(0.15+0.84e^{-\frac{\theta }{15.5}}\right)}^{2}}{M}+0.04^{2}}\;\;\theta >35\\ \sqrt{\frac{{\left(0.24\right)}^{2}}{M}+0.04^{2}}\;\;\theta \leq 35 \end{array}\right.$ [8]	
$\tau_{gnd}$ (Time constant of the ground multipath)	6 s [19]	
$\tau_{air}$ (Time constant of the airborne multipath)	7 s [19]	

^1^ GND: Ground; ^2^ GAD: Ground Accuracy Designator.

**Table 3:** Number of critical satellites for GAST D1 for GPS L1 and Galileo E1 signals.

Number of Satellites in View	GAST D1 with GPS L1				Number of Satellites in View	GAST D1 with Galileo E1			
	DH = 200 ft to Threshold		Threshold to Roll Out			DH = 200 ft to Threshold		Threshold to Roll Out	
	$\sigma_{G}$ (mm/km)					$\sigma_{G}$ (mm/km)			
	4	8	4	8		4	8	4	8
4	4 (by definition)				4	4 (by definition)			
5	2.4430	2.9772	2.2769	2.6091	5	NA	NA	NA	NA
6	0.8113	0.9266	0.7658	0.8652	6	0.0000	0.0000	0.0000	0.0000
7	0.2095	0.2663	0.1903	0.2436	7	0.0010	0.0010	0.0010	0.0010
8	0.0801	0.1092	0.0722	0.1001	8	0.0050	0.0277	0.0033	0.0201
9	0.0535	0.0711	0.0502	0.0661	9	0.0000	0.0000	0.0000	0.0000
10 or more	0.0000	0.0000	0.0000	0.0000	10 or more	0.0000	0.0000	0.0000	0.0000

**Table 4:** Number of critical satellites for GAST E ($\sigma_{G}=$ 4 mm/km for SF).

Number of Satellites in View	GAST E, DC (GPS/Galileo SF, DF)				Number of Satellites in View	GAST E, SC (GPS SF, DF)			
	DH = 200 ft to Threshold		Threshold to Roll Out			DH = 200 ft to Threshold		Threshold to Roll Out	
	DF	SF	DF	SF		DF	SF	DF	SF
5	5 (by definition)				4	4 (by definition)			
6 to 12	0.0000	0.0000	0.0000	0.0000	5	3.9902	1.3485	3.9055	1.3257
13	0.0000	0.0000	0.0000	0.0000	6	1.6407	0.1654	1.5496	0.1533
14	0.0000	0.0000	0.0000	0.0000	7	0.5711	0.0232	0.5304	0.0220
15 or more	0.0000	0.0000	0.0000	0.0000	8	0.2563	0.0031	0.2359	0.0026
					9	0.1825	0.0021	0.1720	0.0019
					10 or more	0.0078	0.0000	0.0063	0.0000

**Table 5:** Number of critical satellites for GAST E ($\sigma_{G}=$ 8 mm/km for SF).

Number of Satellites in View	GAST E, DC (GPS/Galileo SF, DF)				Number of Satellites in View	GAST E, SC (GPS SF, DF)			
	DH = 200 ft to Threshold		Threshold to Roll Out			DH = 200 ft to threshold		Threshold to Roll Out	
	DF	SF	DF	SF		DF	SF	DF	SF
5	5 (by definition)				4	4 (by definition)			
6 to 12	0.0000	0.0000	0.0000	0.0000	5	3.9902	1.9088	3.9055	1.8860
13	0.0000	0.0000	0.0000	0.0000	6	1.6407	0.5491	1.5496	0.5237
14	0.0000	0.0000	0.0000	0.0000	7	0.5711	0.1230	0.5304	0.1156
15 or more	0.0000	0.0000	0.0000	0.0000	8	0.2563	0.0614	0.2359	0.0578
					9	0.1825	0.0489	0.1720	0.0461
					10 or more	0.0078	0.0000	0.0063	0.0000

## Data Availability

The research data used within the SESAR project is not for public use.

## Appendix A

This appendix derives the standard deviation of the smoothed noise after applying the carrier-smoothing filter. The smoothed noise after applying the carrier-smoothing filter can be expressed as the following equation:

(A1) $$y\left(k\right)=Ay\left(k-1\right)+(A-1)x(k).$$

The symbols x and y represent the input white Gaussian noise with the standard deviation $\sigma_{raw}$ and the smoothed noise. The epoch index is denoted as k, and the constant A is defined as $1-\frac{T_{s}}{\tau_{sm}}$, where the terms $T_{s}$ and $\tau_{sm}$ indicate the sampling interval and the smoothing time constant. In addition, $\frac{T_{s}}{\tau_{sm}}$ is defined as K. At any epoch k, the smoothed noise $y\left(k\right)$ can be alternately expressed by x(k) as the following equation:

(A2) $$y\left(k\right)=\left(A-1\right)\sum_{j=1}^{k}A^{k-j}x(k).$$

Using Equation (A2), the variance of the smoothed noise $y\left(k\right)$ can be calculated as the following equation:

(A3) $$E\left[y{\left(k\right)}^{2}\right]=\sigma_{WGN-smooth}^{2}(k)={\left(A-1\right)}^{2}\sum_{j=1}^{k}A^{2(k-j)}\sigma_{raw}^{2}.$$

We assume that the noise terms at different epochs are not correlated. Equation (A3) can be further simplified as the following:

(A4) $$\sigma_{WGN-smooth}^{2}(k)={\left(A-1\right)}^{2}\sigma_{raw}^{2}\frac{1-A^{2k}}{1-A^{2}}.$$

For infinite k, the standard deviation of the smoothed noise can be expressed as the following:

(A5) $$\sigma_{WGN-smooth}^{2}={\left(A-1\right)}^{2}\sigma_{raw}^{2}\frac{1}{1-A^{2}}.$$

Since A−1 can be replaced by $\frac{1}{K}$, Equation (A5) gives the same expression as Equation (20).
